# First Evaluation of Infrared Thermography as a Tool for the Monitoring of Udder Health Status in Farms of Dairy Cows

**DOI:** 10.3390/s18030862

**Published:** 2018-03-14

**Authors:** Mauro Zaninelli, Veronica Redaelli, Fabio Luzi, Valerio Bronzo, Malcolm Mitchell, Vittorio Dell’Orto, Valentino Bontempo, Donata Cattaneo, Giovanni Savoini

**Affiliations:** 1 Department of Human Sciences and Quality of Life Promotion, Università Telematica San Raffaele Roma, Via di Val Cannuta 247, Rome 00166, Italy; 2 Department of Veterinary Medicine, Università degli Studi di Milano, Via Celoria 10, Milan 20133, Italy; veronica.redaelli@unimi.it (V.R.); fabio.luzi@unimi.it (F.L.); valerio.bronzo@unimi.it (V.B.); 3 Animal & Veterinary Sciences, Scotland’s Rural College, Roslin Institute Building, Easter Bush, Midlothian EH25 9RG, UK; malcolm.mitchell@sruc.ac.uk; 4 Department of Health, Animal Science and Food Safety (VESPA), Università degli Studi di Milano, Via Celoria 10, Milan 20133, Italy; vittorio.dellorto@unimi.it (V.D.); valentino.bontempo@unimi.it (V.B.); donata.cattaneo@unimi.it (D.C.); giovanni.savoini@unimi.it (G.S.)

**Keywords:** dairy cow, udder health status, mastitis detection, infrared thermography, imaging analysis

## Abstract

The aim of the present study was to test infrared thermography (IRT), under field conditions, as a possible tool for the evaluation of cow udder health status. Thermographic images (n. 310) from different farms (n. 3) were collected and evaluated using a dedicated software application to calculate automatically and in a standardized way, thermographic indices of each udder. Results obtained have confirmed a significant relationship between udder surface skin temperature (USST) and classes of somatic cell count in collected milk samples. Sensitivity and specificity in the classification of udder health were: 78.6% and 77.9%, respectively, considering a level of somatic cell count (*SCC*) of 200,000 cells/mL as a threshold to classify a subclinical mastitis or 71.4% and 71.6%, respectively when a threshold of 400,000 cells/mL was adopted. Even though the sensitivity and specificity were lower than in other published papers dealing with non-automated analysis of IRT images, they were considered acceptable as a first field application of this new and developing technology. Future research will permit further improvements in the use of IRT, at farm level. Such improvements could be attained through further image processing and enhancement, and the application of indicators developed and tested in the present study with the purpose of developing a monitoring system for the automatic and early detection of mastitis in individual animals on commercial farms.

## 1. Introduction

In dairy farming, the monitoring of udder health status plays an important role in the production of milk [1]. Mastitis is the most frequent disease that can affect the health status of the udder and the quantity and quality of milk yielded. Its early detection, thought an effective and automatic monitoring, can be a way to improve the farm efficiency [2]. Many indicators, methods, and devices have been developed to reach this goal. Some of these, such the somatic cell count (*SCC*), the bacteriological examination of milk samples (BAC), the DeLaval Cell Counter (DCC) or the California Mastitis Test (CMT) are laboratory analysis, or portable devices, not routinely applicable in the monitoring of animals’ health on-farm due to the costs and time requirements [3]. Others are indicators that can be evaluated at farm level, like the electrical conductivity [4,5,6] or the aspect of milk [7]. Nevertheless, these are indirect measurements of the infection and their abilities and sensitivities to detect all cases, in particular when subclinical, are not always sufficient [2]. For this reason, new indicators and technologies should be considered.

When a clinical mastitis occurs, some systemic or local signs may be observed. An acute-phase response and local inflammation can result in fever in the animals [8] and changes in blood flow at tissue level [9] that can be the origin of an increase of the udder skin surface temperature (USST) [10,11,12]. This change can be detected by an infrared thermography (IRT) in a non-invasive and contact-less way. The physical principal at the basis of the IRT is described by the Planck, Wien and Stefan-Boltzmann laws [13]. A body that has a temperature higher than the absolute zero, emits electromagnetic radiation in the infrared (IR) spectrum. The relationship between the energy emitted by the body surface, the wave length of this radiation and the temperature of the body is mathematically described. This radiation can be detected through a sensor array and used to build a thermographic image where the intensity, or the color, of each pixel is proportional to the corresponding temperature of the surface observed [14,15].

Infrared thermography has been already investigated by some authors as a possible tool for the monitoring of udder health status. Scott et al. (2000) [16] found that quarters artificially challenged by *E. coli* lipopolysaccharide (LPS) showed an increase of the USST of circa 2.3 °C after 6 h from the treatment. Temperature increments were measurable in the maximum and average temperatures of the whole udder (*T_max_*, *T_avg_*) and they were not observable in the quarters not challenged. Hovinen et al. (2008) [2], in a similar study carried out on 6 cows found that IRT was able to detect temperature rises, from 1 to 1.5 °C, in left forequarters affected by artificially induced, clinical mastitis. Udder skin temperatures increased after 4 hours following the infusion of LPS and the USST was linearly related to the animals’ rectal temperature (T_R_). Metzner et al. (2015) [17], in a study where right hindquarters of five cows were experimentally infected by *E. coli*, found that IRT was useful to detect induced mastitis when *T_max_* values, of one or both hindquarters, were evaluated with an interval of no more than two hours between examinations. Colak et al. (2008) [11], in a study performed on 94 dairy cows, found a strong correlation (r = 0.92) between the USST, measured by IRT, and the quarter health status evaluated performing the California Mastitis Test (CMT). In a similar study, carried out by Polat et al. (2010) [10] with an experimental group of 62 dairy cows, the accuracy of IRT in the detection of subclinical mastitis (*SCC* > 400,000 cells/mL) was evaluated and compared with CMT. Results showed a positive correlation between the USST and the CMT score (r = 0.86) and the accuracy of a possible IRT test reached levels of sensitivity and specificity of 95.6% and 93.6%, respectively in line with the values determined by the CMT (that were of 88.9% and 98.9%, respectively). However, all these experiments were conducted on few animals, or cases, with different settings in terms of: quarters investigated, areas of the udder considered for the thermographic evaluation of the skin, temperature indicators considered (such as the *T_max_*, *T_avg_*, differences between quarters, before and after infections, etc.), environmental variables (such as atmospheric temperature, humidity, etc.) and definition of the udder health state (*SCC*, CMT, etc.). Furthermore, additional negative or incongruent results have been reported by other authors [12,18,19]. For example, Pezeshki et al. (2011) [19], in a study that involved nine primiparous Holstein Friesian cows, artificially infected by *E. coli* in their left quarters, found that a thermal camera was able to detect changes of 2–3 °C in the USST. However, these changes were detected only when clinical signs of mastitis were visible. Therefore, the authors concluded that IRT was not a useful technique to detect a possible case of *E. coli* mastitis in an early stage. Bortolami et al. (2015) [12], in a study carried out with the aim to evaluate the use of IRT to detect a subclinical mastitis, reported a low diagnostic ability of the thermography. A group of 98 Holstein Friesian cows were involved in the study; *SCC* and bacteriological culture of milk samples were considered. Results showed a not significant association between etiological agents and USSTs. Nevertheless, the authors found a significant relationship between the levels of somatic cell score (SCS), calculated as a logarithmic transformation of *SCCs*, and the udder surface temperatures. On the basis of this relationship they concluded that IRT could be a screening tool useful for the evaluation of udder inflammation status. Berry et al. (2003) [18], in a study that involved ten multiparous Holstein Friesian cows, investigated the variations of USSTs in a daily and within-day scale with the aim to improve a base knowledge for the development of future methods for the early detection of a possible case of mastitis. The IRT was used for the measurement of udder surface temperatures and possible effects, of environmental factors, were also investigated. Results have shown that a model, based on USST values of previous days, could predict the current udder temperature. Furthermore, residual values not predicted by the model were less than 0.5 °C. This suggested that possible rises of USST, related to a case of mastitis, could be potentially detected by IRT. Nevertheless, to achieve a good prediction accuracy, the model needed to consider some environmental parameters. This fact led the authors to suggest that new experiments, on different field conditions, are necessary as also remarked by many other authors [2,10,11]. Furthermore, efficient technologies must be developed if the final target is an automation of the evaluation of USST by thermography [17,18].

The aim of the present study was, therefore, a further evaluation of the IRT, under field condition, considering a greater number of dairy cows, from different farms, reared in different ambient conditions. A significant number of thermographic images were analyzed and two different settings to classify udder health status were investigated. Some dedicated imaging elaborations were developed and new thermographic indices were calculated having in mind the use of IRT in a possible automatic system. Finally, the accuracy of this technology was evaluated in order to determine its applicability and effectiveness as a primary screening tool in cow udder health.

## 2. Material and Methods

### 2.1. Animals and Farms

The study was carried out in January 2018. It involved 155 dairy cows reared in three different medium size farms located in the Lombardy region (North of Italy). In detail, the experimental group was composed by: 92 Holstein Friesian cows of the farm 1; 35 Holstein Friesian cows of the farm 2 and 28 Holstein Friesian cows of the farm 3. Each cow did not have any clinical sign of mastitis. Stage of lactation was different for each cow and ranged between 15 to 275 days. Cows were feed twice a day with a total mixed ration according to their state of lactation. They were housed in free-stalls with straw beddings changed 2 or 3 times per week. Cows were milked by automatic milking systems (AMS) of different manufacturers and using specific settings for each farm. In detail, they were: one BouMatic MR-D1 (BouMatic LLC Stoughton Rd, Madison, WI, USA) for farm 1; one Fullwood Merlin M^2^ (Fullwood Ltd., Grange Road, Ellesmere, Shropshire, UK) for farm 2; and one BouMatic MR-S1 for farm 3. In each farm, during the time of the experiments, neither ventilators nor water dropping were active.

### 2.2. Milk Sampling, Milk Sample Analysis and Definition of Udder Health Status

Immediately before to acquire thermografic images, each mammary gland was examined in order to avoid a possible case of clinical mastitis. A clinical mastitis was diagnosed when milk from one or more glands was abnormal in color, viscosity, or consistency; with or without accompanying signs of heat, pain, or redness. Clinical signs of mastitis was assessed according to National Mastitis Council guidelines [20]. From each cow, a composite milk sample of all udder quarters was automatically collected by the milking robot. At the end of the day of sampling, a technician of the Regional Breeders Association (Associazione Regionale Allevatori Lombardia—ARAL), within a Dairy Herd Improvement Program (DHI), collected milk samples of the farm that were processed for *SCC* in the following day of sampling, following international recommendations [21]. 

In accordance with experimental designs used in similar studies [10], *SCC* results were considered to classify the health status of udders. Since different thresholds are generally adopted to classify subclinical mastitis [22,23], two cutoffs were taken into consideration in order to discriminate healthy versus not healthy cases: 200,000 cells/mL and 400,000 cells/mL.

### 2.3. Thermographic Images Collection

**Figure 1:**
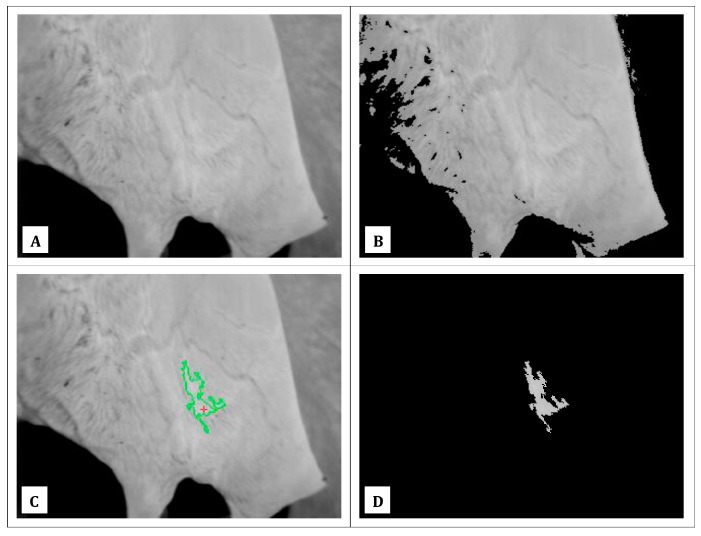
The figure shows some image elaborations performed by the algorithm of the developed software application. In details, in (**A**) is reported an example of a thermographic image acquired during the study carried out; In (**B**) is shown the result obtained applying as thresholds, a range of intensities calculated through the above reported formulas ([1,2]) and after identifying in thermographic image the pixel with the maximum intensity value (*PI_max_*). In the figure, almost the whole cow udder is highlighted. As a consequence, a grid, of dimensions 4 × 4 pixels can be applied in order to calculate the surface distribution of temperatures. In a following step, the maximum value of udder skin temperature (*T_max_*) can be identified as the maximum value within the surface temperatures calculated; In (**C**), it is shown with a red cross the location of the pixel *PI_max_* and with a green contour the *AP_T_* calculated; In (**D**), finally, is reported the “temperatures proximity area” (*AP_T_*) obtained considering the coordinates of *PI_max_* and a set of connected pixels which intensities are different from zero after applying the above reported filter [3].

Thermographic images were collected using a commercial infrared camera (Thermo GEAR-G120 EX-Nippon Avionics Co., Tokyo, Japan). It had an uncooled detector focal plane array (microbolometer) with a resolution of 320 × 240 pixels. Its accuracy was ±2 °C with a sensitivity of 0.04 °C (at 30 °C) while its physical dimensions were 21.2 cm × 7.5 cm × 13.8 cm (H × W × D). Prior to image acquisition, the ambient temperature of milking parlor was recorded and used to allow internal compensation for this parameter (i.e., calibration) by thermal imaging camera algorithms. The range of ambient temperature recorded during the experimental period was from 6 to 10 °C with a mean value of 8 °C. The camera operator ensured optimum image focus during image acquisition. An emissivity of 0.98 was employed throughout in accordance with previously published studies carried out on cow udders [2,24,25,26,27,28,29]. Thermographic images were captured positioning the camera at udder level, at a distance of circa 0.6 m [10,28,30,31] from each udder side (collecting as a result, two thermographic images for each udder—Figure 1A). During thermographic images acquisition, the camera operator acquired at least three images for each udder side. This step guaranteed to have, for the analysis that followed, one clear image without any movement of the cow or leg that could partially hide the udder. Only one set of thermographic images (i.e., the right and the left side of each udder) was acquired for each animal involved in the experiment. Thermographic images were acquired just before the start of milking procedures having in mind a possible future automation of the use of USST as an indicator for the monitoring of udder health status.

All acquired images were analyzed by a dedicated software application developed in LabVIEW (National Instruments, Austin, TX, USA—version: 8.5) using also some specific subroutines of the Vision Acquisition Software (NI—version: 2009) and Vision Development Module (NI—version: 2009). The software application was able to work, off-line, on a set of “.bpm” files [32] formatted with a gray-level scale and a resolution of 8 bit. On each image file, the software algorithm performed the following tasks in automatic:

- It identified the pixel with the maximum intensity (*PI_max_*, Figure 1C), calculating its coordinates inside the image and its value (equal to the maximum recorded temperature in the thermographic image).
- It calculated a range of intensities to use as thresholds, according to the following formulas:
  (1) $$Upper\;value=PI_{max}$$
  (2) $$Lower\;value=0.75\times PI_{max}$$
  These values were selected considering both, the average USSTs that have been observed in previous experiments [2,10,11,16,19] and the increments that have been reported in case of subclinical and clinical mastitis. The range of intensities calculated was applied as a filter [33], to the thermographic image, in order to detect the udder of the cow (Figure 1B).
- On the filtered image, it applied a grid made by image subsections of 4 × 4 pixels.
- On each image subsection, it calculated the pixel average intensity (i.e., the recorded average temperature of the image subsection evaluated).
- On the resulting set of pixel average intensities, it selected the maximum value and it considered that number as the recorded maximum temperature of the thermographic image evaluated (i.e., the *T_max_*), taken as possible index of the udder health status in accordance with results of previous scientific studies [2,17]).
- It calculated a “temperature proximity area” (*AP_T_*, Figure 1C,D) considering the coordinates of *PI_max_* as a starting point and a set of connected pixels which intensities were different from zero after applying the following filter:
  (3) $$PI_{New}=\{\begin{array}{cc} 0 & PI_{Old}<PI_{max}-T\\ PI_{Old} & PI_{max}-T\leq PI_{Old}\leq PI_{max} \end{array}$$
  where the indicator *T* (tolerance) was set up at a level of 15 [34]. This last value was selected considering the increments of USST already found in scientific literature [2,10,11,16,19]. Furthermore, a connectivity mode of 4 pixels was used for the recursive application of the above reported filter [35]. This value specified at the algorithm whether a pixel should be considered in the following cycle. In detail, it imposed at the software application to take into consideration the pixels that were at the cardinal points (i.e., North, East, South and West) of the pixel under evaluation for the filtering operations that followed.

After evaluation of all acquired thermographic images, the software application reported the results in a “.txt” file in order to allow statistical analysis. At this purpose, for each udder, only the thermographic image that showed the highest value of USST was studied as a possible method for the monitoring of udder health status.

### 2.4. Statistical Analysis

Data obtained from image elaborations were investigated through statistical analysis performed using the “R” software tool (version 3.4.3, 2017) [36]. The relationships between dependent statistical variable *T_max_* and independent statistical variables *SCC* and *AP_T_* were studied. The following linear model was fitted:

(4) $$Y_{ijk}=\beta_{o}+\beta_{1}\;SCC_{i}+\beta_{2}\;AP_{T_{j}}+\beta_{3}\;SCC\times AP_{T_{k}}+e_{ijk}$$

where: *Y_ijk_* were the values of variable *T_max_* calculated from thermographic images evaluated; *SCC_i_* (log-transformed) was the effect of somatic cell counts performed on milk samples collected; *AP_Tj_* was the effect of “temperature proximity areas” calculated on thermographic images investigated; (*SCC* × *AP_Tk_*) was the effect of the first order interaction between somatic cell counts and “temperature proximity areas” considered; and *e_ijk_* were the residual errors. To calculate the values of model’ linear coefficients ($\beta_{n}$), and to evaluate their significance, a linear regression analysis was performed using the procedure “*lm*” of the package “*stats*” (version 3.4,3—[37,38]).

In a following phase of statistical analysis, the ability of the variable *T_max_* to discriminate a possible case of mastitis was investigated. When *T_max_* overcame a defined threshold, a case of mastitis was supposed (i.e., the result of a possible statistical test was set up as positive). On the basis of *SCC* performed on the corresponding milk sample, udder health status was defined (i.e., “healthy” if *SCC* was lower than a defined threshold or “not healthy” if *SCC* was higher than the selected threshold). The results of statistical test, and of udder health status definitions, were compared and classified as following: true positive (*TP*), when the statistical test was able to detect a case of healthy udder; false positive (*FP*), when the statistical test highlighted a possible case of mastitis evaluating a case of healthy udder; true negative (*TN*), when the statistical test correctly classified a case of not healthy udder and false negative (*FN*), when a not healthy udder was not detected by the statistical test. When all results were classified, the performance of the statistical test based on the evaluations of *T_max_* was calculated as: sensitivity and specificity, in accordance with the following formulas:

(5) $$Sensitivity\left[\%\right]=\frac{TP}{FN+TP}$$

(6) $$Specificity\left[\%\right]=\frac{TN}{FP+TN}$$

Of course, the statistical test gave different couples of sensitivity and specificity for each possible threshold used to evaluate the variable *T_max_*. For this, a receiver operating characteristic (ROC) curve was build using the procedures “*prediction*” and “*performance*” of the package “*ROCR*” (version 1.0.7—[39]). Analyzing the curve, a specific cutoff was selected and the corresponding couple of sensitivity and specificity was identified as final performance reached by the variable *T_max_* in the detection of a possible case of an udder with high *SCC*. Furthermore, the area under the curve (AUC) was also considered to study the performance of the variable *T_max_*; and both definitions of udder health status were investigated (i.e., less than 200,000 cells/mL and less than 400,000 cells/mL). 

## 3. Result

In a first phase of statistical analysis the relationships between: the variable *T_max_*; the values of *SCC* carried out on milk samples collected; and the values of *AP_T_* obtained from the image elaborations performed on thermographic images collected; were studied. A linear regression was conducted and the values of linear coefficients were estimated. In Table 1, these values, and their significances, are reported.

**Figure 2:**
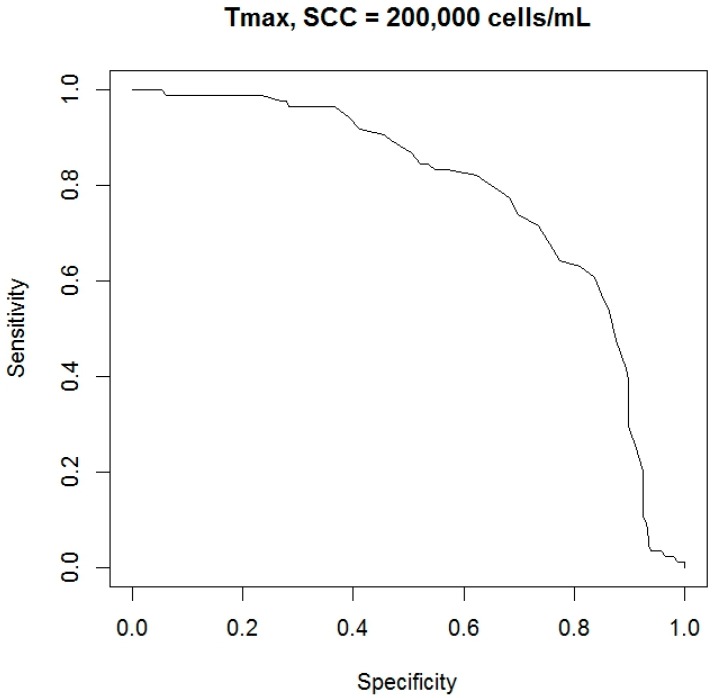
Receiver operating characteristic (ROC) curve of the statistical test built evaluating the variable *T_max_* and different possible cutoff levels. For the determination of udder health status, an *SCC*’ threshold of 200,000 cells/mL was used. The ROC curve was obtained through the procedures “*prediction*” and “*performance*”, package “*ROCR*” of the “R” statistical software tool.

**Figure 3:**
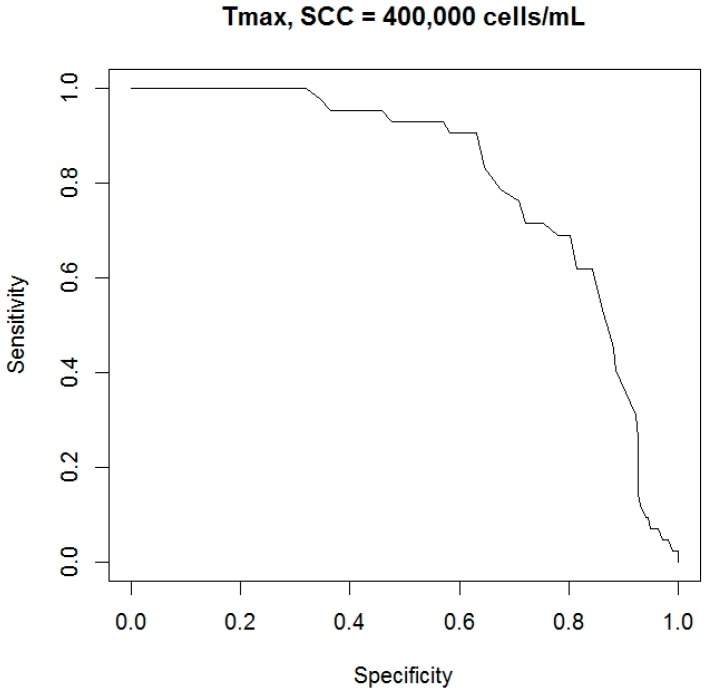
ROC curve of the statistical test built evaluating the variable *T_max_* and different possible cutoff levels. For the determination of udder health status, an *SCC*’ threshold of 400,000 cells/mL was used. The ROC curve was obtained through the procedures “*prediction*” and “*performance*”, package “*ROCR*” of the “R” statistical software tool.

In a following phase of statistical analysis, the detection performances of the variable *T_max_* were investigated. Two different thresholds of *SCC* were used to classify the udders health status (i.e., 200,000 cells/mL and 400,000 cells/mL). For each *SCC*’ threshold, a ROC curve was calculated through the couples of sensitivity and specificity shown by the statistical test when different possible cutoff levels were selected. In Figure 2 and Figure 3, the ROC curves obtained are reported.

In a final phase of statistical analysis, the AUC and the final cutoff level were calculated for each ROC curve obtained. Final cutoff levels were identified considering the point, in the curve, closer to the best theoretical result (i.e., the point in the graph in the upper right corner equal to a sensitivity and specificity of 100%). Results obtained are reported in Table 2 while in Table 3 means and standards errors values of the main indicators investigated, for each criterion adopted to classified udder health status, are also shown.

## 4. Discussion

The relationship between the levels of somatic cell and the values of *T_max_*, calculated on the thermographic images collected during this study, showed to be significant. Furthermore, when the levels of somatic cells increase, the values of *T_max_* follow the same trend. Similar results have been found by other authors. Barth et al. [40], in a study conducted on 6 cows followed for 8 days, found that USST increase when measured on quarters characterized by *SCC* higher than 100,000 cell/mL (34.1 °C Vs. 33.6 °C). Polat et al. [10], in a study curried out on 62 dairy cows, found a positive correlation between USST values and *SCC*. When *SCC* increased, the values of USST showed to increase logarithmically and the best linear model that fitted this relationship reached an R^2^ value of 0.73. Martins et al. [41], in a study performed on 37 ewes, found that higher USSTs were related to high *SCC*. Therefore, even though bacteriological analysis were not performed on milk samples collected in our study, statistical results seem to confirm that USST could be a possible index for the detection of a not healthy state of cow udder.

In our study, also the area of cow udder involved in a local increase of USST was investigated calculating in each thermographic image what we called a “temperature proximity area” (*AP_T_*). This variable, never considered in a previous published paper, has shown a significant relationship with the trend of *T_max_* (and of *SCC*). When values of *AP_T_* showed a decrease (and levels of *SCC* an increase) the values of *T_max_* showed to be higher. This result suggests that high USSTs, to be significant, should be always coupled with small values of *AP_T_* in order to highlight a real local rise of temperature due to changes of underlying circulation and tissue metabolism [18], both caused by the presence of a local inflammation of the mammary gland [11]. This variable, therefore, could be useful to increase the detection performance of a monitoring system based on the use of the variable *T_max_*. In previous studies, in fact, *T_max_* has been measured through the pixel of maximum intensity found in the thermographic image evaluated [17]. With this procedure, the accuracy of the measurement could be affected by a possible error of the thermographic sensor (due by noise, etc.). In our study, we tried to limit this effect not considering a single spot for the measurement of *T_max_* but an average value calculated on a small image section of dimensions 4 x 4 pixels. Nevertheless, this methodology does not avoid other possible reading errors due by the use of an infrared camera. It is well known, in fact, that commercial infrared cameras can show reading errors up to ±2 °C [29]. Even though most of them have an internal function to automatically perform a sensor calibration, this effect could negatively affect the performance of a system for the automatic monitoring of udder health status based on the evaluation of the variable *T_max_* through an absolute threshold. Furthermore, also the effect of each animal could negatively affect the use of this index in a field application. Thus, a combined evaluation of the variables *T_max_* and *AP_T_* should allow to overcome the above cited issues and to reduce possible false positive cases. Another reason that could promote the use of a combined evaluation of these variables could be a better classification of cases where the *PI_max_* is detected outside the udder surface (such for example in the hairless area of the adjacent leg). These cases generally happen when a significant rise of USST is not locally present in the udder and are often coupled with a high value of the variable *AP_T_*. Therefore, a combined evaluation of *T_max_* and *AP_T_* could allow a better management of these cases and so to permit the use of USST, in field conditions, for the automatic monitoring of udder health status. Future studies would be useful to confirm this hypothesis.

In previous studies some authors have investigated which area of the cow udder should be more promising for the measurement of a correct and significant USST. Porcionato et al. [42], for the measurement of the USST, used three areas selected considering the udder’ height in the dorsal-ventral direction. Hovinen et al. [2] considered circles of dimensions 40 × 40 pixels positioned immediately above the teats from the lateral side of the cow udder reporting that the maximum temperature recordable on each udder not always was inside the area considered for the measurements. Pezeshki et al. [19], used two rectangles of dimensions 25 × 25 pixels positioned above the teats, in the rear side of the udder. Metzner et al. [43], in order to limit possible mistakes due by personal interpretation of thermographic images, tried to define a standardized procedure based on the manual drawing of three different geometrical shapes on the rear side of each cow udder. These geometrical shapes were: a polygon, two rectangles, and two lines built applying specific rules to the sizes of each evaluated quarter. The authors have remarked that an effective detection of mastitis, in a milking parlor, through an automatic monitoring system, could be reached only if the USST would be measured and analyzed in a standardized way and they suggested as main features of a possible algorithm: (1) an automatic identification of the udder shape based on the major temperatures of this area; (2) the elimination of the anatomical parts not useful to the measurement of USST; and (3) the use of all possible pixels of the thermographic image in order to limit possible measurement mistakes due by dirt particles on udder skin and/or other possible imaging artifacts. In our study, an algorithm for the calculation of USST from thermographic images was developed in accordance with suggestions provided by Metzner et al. [43]. The algorithm allowed to detect, automatically, the udder surface on the basis of the higher temperatures of udder skin and to calculate the USST considering the maximum number of pixels of the thermographic image evaluated. This algorithm could run in real time and it could be easily integrated in a real monitoring system for the udder health status surveillance. Thus, it could be considered as a possible technical solution to the needs previously stated by other authors. Further experiments would be useful in order to test also this technology solution in a real field application.

However, in the present study, *T_max_* values were also investigated to discriminate possible not healthy states of cow udder. Two different *SCC*’ thresholds were used to classify the udder health status. Results obtained showed as a detection accuracy of this indicator: a sensitivity of 78.6% and a specificity of 77.9%, using as *SCC*’ threshold a level of 200,000 cells/mL; a sensitivity of 71.4% and a specificity of 71.6% using as *SCC*’ threshold a level of 400,000 cells/mL; and values of AUC of ROC curves that were in the limit of good and fair diagnostic accuracy (0.80 and 0.81, respectively). Scientific researchers that have studied the performance of the variable USST for the detection of a possible case of not healthy mammary gland are really few. In a study that involved 62 dairy cows, Polat et al. [10] reported, as detection performance of the USST, a sensitivity of 83.5% and a specificity of 100% with an *SCC* threshold of 200,000 cells/mL and a cutoff of 34.7 °C; and a sensitivity of 95.6% and a specificity of 93.6% considering an *SCC* threshold of 400,000 cells/mL and always a cutoff of 34.7 °C. In a study that involved 65 dairy camels, Samara et al. [7] reported as accuracy of the index USST a sensitivity of 89% and a specificity of 96%, having considered an *SCC* threshold of 432,000 cells/mL and a cutoff value of 35.7 °C. Our results were lower than those cited above. However, in our study, many animals have been considered to collect thermographic images in a field condition. Furthermore, *SCCs* were evaluated on milk samples composed by all udder quarters of each cow. As a consequence, possible rises of *SCCs*, due by mastitis cases, may have been partially masked by dilution effects. Thus, we think that our results can be considered as acceptable as a first field application of this technology and we think that they confirm what was reported by other authors about the use of USST as a possible index for the rapid and noninvasive evaluation of udder health status [2,10,11,12,14,17,18,41,43,44]. Nevertheless, further studies would be useful in order to reach, also at farm level, a better accuracy in the automatic and early detection of possible cases of mastitis.

## 5. Conclusions

The variable USST showed a significant relationship with the classes of *SCC* confirming that it could be a useful index for the early detection of a possible case of not healthy cow udder. The sensitivity and specificity found, considering two different classes of *SCC* as thresholds to classify a possible state of not healthy cow udder, were lower than those reported by other authors. Nevertheless, they were acceptable considering the large number of animals involved in the present experiment and the range field conditions during the study even though no validation of true cases was done by sample culture or repetition in different days. Future experiments will facilitate improvements in the use of IRT and the development of a monitoring system for the automatic and early detection of mastitis, in individual animals of commercial farms, also considering the image processing and the indicators developed and tested in the present study.

## Figures and Tables

**Table 1:** Values and significance of linear coefficients used to study the relationships between the dependent variable *T_max_* (i.e., the maximum temperature of the thermographic image evaluated) and the independent variables: *SCC* (somatic cell count) and *AP_T_* (i.e., the “temperature proximity area”). In the linear model, the first order interaction between *SCC* and *AP_T_* was also included. Values and significance of linear coefficients were estimated through the procedure “*lm*”, package “*stats*” of the “R” statistical software tool.

Items	Linear Coefficients		
	Estimate	Standard Error	Significance
Intercept	33.6	0.98	*p* < 0.01
*SCC* (log)	0.881	0.0430	*p* < 0.05
*AP_T_*	−0.000995	0.0000395	*p* < 0.05
*SCC* × *AP_T_*	0.000369	0.0000201	*p* < 0.05

**Table 2:** Final performance of the statistical test based on the evaluation of the variable *T_max_*. In the table, area under the curve (AUC), sensitivity, specificity, and the corresponding cutoff level are reported for each *SCC* threshold used to classify udders health status. The values reported in the table were calculated through a customized function developed for the “R” statistical software tool.

*SCC* Threshold (Cells/mL)	AUC (Area)	Sensitivity (%)	Specificity (%)	Cutoff Level (°C)
200,000	0.805	78.6	77.9	35.1
400,000	0.811	71.4	71.6	35.3

**Table 3:** Descriptive statistics of the main indicators investigated (*T_max_*, *SCC* and *AP_T_*) in terms of mean and standard error (S.E.) values for each criterion adopted to classify the udder health status (i.e., criterion 1: udder health = “healthy” if *SCC* < 200,000 cells/mL; criterion 2: udder health = “healthy” if *SCC* < 400,000 cells/mL).

*SCC* Threshold (Cells/mL)	Udder Health State (Healthy/Not Healthy)	Cases (n)	*T_max_* (°C, Means ± S.E.)	*SCC* (×10^3^ Cells/mL, Means ± S.E.)	*AP_T_* (# Pixels, Means ± S.E.)
200,000	healthy	113	34.19 ± 0.17	62.64 ± 4.53	2460 ± 90
	not healthy	42	35.79 ± 0.15	592.38 ± 71.40	1476 ± 151
400,000	healthy	134	34.40 ± 0.16	92.62 ± 7.33	2397 ± 85
	not healthy	21	36.08 ± 0.22	930.81 ± 96.58	898 ± 79

## References

[1] Zaninelli M., Rossi L., Costa A., Tangorra F.M., Agazzi A., Savoini G. (2015). Monitoring of goats’ health status by on-line analysis of milk electrical conductivity. Large Anim. Rev..

[2] Hovinen M., Siivonen J., Taponen S., Hänninen L., Pastell M., Aisla A., Pyörälä S. (2008). Detection of Clinical Mastitis with the Help of a Thermal Camera. J. Dairy Sci..

[3] Pyörälä S., Taponen S. (2009). Coagulase-negative staphylococci-emerging mastitis pathogens. Vet. Microbiol..

[4] Zaninelli M., Rossi L., Costa A., Tangorra F.M., Agazzi A., Savoini G. (2015). Signal spectral analysis to characterize gland milk electrical conductivity in dairy goats. Ital. J. Anim. Sci..

[5] Zaninelli M., Tangorra F.M., Costa A., Rossi L., Dell’Orto V., Savoini G. (2016). Improved fuzzy logic system to evaluate milk electrical conductivity signals from on-line sensors to monitor dairy goat mastitis. Sensors.

[6] Zaninelli M., Agazzi A., Costa A., Tangorra F.M., Rossi L., Savoini G. (2015). Evaluation of the fourier frequency spectrum peaks of milk electrical conductivity signals as indexes to monitor the dairy goats’ health status by on-line sensors. Sensors.

[7] Samara E.M., Ayadi M., Aljumaah R.S. (2014). Feasibility of utilising an infrared-thermographic technique for early detection of subclinical mastitis in dairy camels (Camelus dromedarius). J. Dairy Res..

[8] Radostits O.M., Gay C.C., Hinchcliff K.W., Constable P.D. (2007). Clinical findings of bovine mastitis. Veterinary Medicine: A Textbook of the Diseases of Cattle, Sheep, Pigs, Goats and Horses.

[9] Paulrud C.O., Clausen S., Andersen P.E., Bjerring M., Rasmussen M.D. (2002). Infrared thermography to evaluate milking induced alterations in teat tissue fluid circulation. J. Dairy Sci..

[10] Polat B., Colak A., Cengiz M., Yanmaz L.E., Oral H., Bastan A., Kaya S., Hayirli A. (2010). Sensitivity and specificity of infrared thermography in detection of subclinical mastitis in dairy cows. J. Dairy Sci..

[11] Colak A., Polat B., Okumus Z., Kaya M., Yanmaz L.E., Hayirli A. (2008). Short Communication: Early detection of mastitis using infrared thermography in dairy cows. J. Dairy Sci..

[12] Bortolami A., Fiore E., Gianesella M., Corrò M., Catania S., Morgante M. (2015). Evaluation of the udder health status in subclinical mastitis affected dairy cows through bacteriological culture, Somatic Cell Count and thermographic imaging. Pol. J. Vet. Sci..

[13] Maldagues X., Moore P. (2001). Nondestructive Testing Handbook, 3rd Edition: Volume 3. Infrared and Thermal Testing.

[14] Poikalainen V., Praks J., Veermäe I., Kokin E. (2012). Infrared temperature patterns of cow’s body as an indicator for health control at precision cattle farming. Agron. Res..

[15] Zaninelli M., Redaelli V., Tirloni E., Bernardi C., Dell’Orto V., Savoini G. (2016). First results of a detection sensor for the monitoring of laying hens reared in a commercial organic egg production farm based on the use of infrared technology. Sensors.

[16] Scott S.L., Schaefer A.L., Tong A.K.W., Lacasse P. (2000). Use of infrared thermography for early detection of mastitis in cows. Agri-Food 2000.

[17] Metzner M., Sauter-Louis C., Seemueller A., Petzl W., Zerbe H. (2015). Infrared thermography of the udder after experimentally induced Escherichia coli mastitis in cows. Vet. J..

[18] Berry R.J., Kennedy A.D., Scott S.L., Kyle B.L., Schaefer A.L. (2003). Daily variation in the udder surface temperature of dairy cows measured by infrared thermography: Potential for mastitis detection. Can. J. Anim. Sci..

[19] Pezeshki A., Stordeur P., Wallemacq H., Schynts F., Stevens M., Boutet P., Peelman L.J., Spiegeleer B.D., Duchateau L., Bureau F. (2011). Variation of inflammatory dynamics and mediators in primiparous cows after intramammary challenge with Escherichia coli. Vet. Res..

[20] Nursing and Midwifery Council (NMC), NMC (National Mastitis Council) (2016). Current Concepts of Bovine Mastitis.

[21] Nursing and Midwifery Council (NMC), NMC (National Mastitis Council) (2017). Laboratory and Field Hand-book on Bovine Mastitis.

[22] De Vliegher S., Laevens H., Opsomer G., Muêlenaere E., De Kruif A. (2001). De Somatic cell counts in dairy heifers during early lactations. Flem. Vet. J..

[23] Chagunda M.G., Larsen T., Bjerring M., Ingvartsen K.L. (2006). L-lactate dehydrogenase and N-acetyl-β-D-glucosaminidase activities in bovine milk as indicators of non-specific mastitis. J. Dairy Res..

[24] Talukder S., Kerrisk K.L., Ingenhoff L., Thomson P.C., Garcia S.C., Celi P. (2017). Infrared technology for estrus detection and as a predictor of time of ovulation in dairy cows in a pasture-based system. Theriogenology.

[25] Montanholi Y.R., Swanson K.C., Schenkel F.S., McBride B.W., Caldwell T.R., Miller S.P. (2017). On the determination of residual feed intake and associations of infrared thermography with efficiency and ultrasound traits in beef bulls. Livest. Sci..

[26] Montanholi Y.R., Odongo N.E., Swanson K.C., Schenkel F.S., McBride B.W., Miller S.P. (2008). Application of infrared thermography as an indicator of heat and methane production and its use in the study of skin temperature in response to physiological events in dairy cattle (*Bos taurus*). J. Therm. Biol..

[27] Weschenfelder A.V., Saucier L., Maldague X., Rocha L.M., Schaefer A.L., Faucitano L. (2013). Use of infrared ocular thermography to assess physiological conditions of pigs prior to slaughter and predict pork quality variation. Meat Sci..

[28] Castro-Costa A., Caja G., Salama A.A.K., Rovai M., Flores C., Aguiló J. (2014). Thermographic variation of the udder of dairy ewes in early lactation and following an Escherichia coli endotoxin intramammary challenge in late lactation. J. Dairy Sci..

[29] McManus C., Tanure C.B., Peripolli V., Seixas L., Fischer V., Gabbi A.M., Menegassi S.R.O., Stumpf M.T., Kolling G.J., Dias E. (2016). Infrared thermography in animal production: An overview. Comput. Electron. Agric..

[30] Sathiyabarathi M., Jeyakumar S., Manimaran A., Jayaprakash G., Pushpadass H.A., Sivaram M., Ramesha K.P., Das D.N., Kataktalware M.A., Prakash M.A. (2016). Infrared thermography: A potential noninvasive tool to monitor udder health status in dairy cows. Vet. World.

[31] Alsaaod M., Syring C., Dietrich J., Doherr M.G., Gujan T., Steiner A. (2014). A field trial of infrared thermography as a non-invasive diagnostic tool for early detection of digital dermatitis in dairy cows. Vet. J..

[32] Zaninelli M., Costa A., Tangorra F.M., Rossi L., Agazzi A., Savoini G. (2015). Preliminary evaluation of a nest usage sensor to detect double nest occupations of laying hens. Sensors.

[33] Zaninelli M., Redaelli V., Luzi F., Bontempo V., Dell’Orto V., Savoini G. (2017). A Monitoring System for Laying Hens That Uses a Detection Sensor Based on Infrared Technology and Image Pattern Recognition. Sensors.

[34] Xiaobo M., Jing Y. Research on object-background segmentation of color image based on LabVIEW. Proceedings of the 2011 IEEE International Conference on Cyber Technology in Automation, Control, and Intelligent Systems (CYBER).

[35] Relf C.G. (2004). Image Acquisition and Processing with LabVIEW.

[36] Team R Core Development (2008). R: A Language and Environment for Statistical Computing.

[37] Wilkinson G.N., Rogers C.E. (1973). Symbolic descriptions of factorial models for analysis of variance. Appl. Stat..

[38] Chambers J.M., Chambers J.M., Hastie T.J. (1992). Linear models. Statistical Models in S.

[39] Sing T., Sander O., Beerenwinkel N., Lengauer T. (2005). ROCR: Visualizing classifier performance in R. Bioinformatics.

[40] Barth K. (2000). Basic investigations to evaluate a highly sensitive infrared-thermograph-technique to detect udder inflammation in cows. Milchwissenschaft.

[41] Martins R.F.S., do Prado Paim T., de Abreu Cardoso C., Stéfano Lima Dallago B., de Melo C.B., Louvandini H., McManus C. (2013). Mastitis detection in sheep by infrared thermography. Res. Vet. Sci..

[42] Porcionato M.A.F., Canata T.F., De Oliveira C.E.L., Santos M.V. (2009). Dos Udder Thermography of Gyr Cows for Subclinical Mastitis Detection/Termografia Do Úbere De Vacas Gir Para Detecção De Mastite Subclínica. Rev. Bras. Eng. Biossistemas.

[43] Metzner M., Sauter-Louis C., Seemueller A., Petzl W., Klee W. (2014). Infrared thermography of the udder surface of dairy cattle: Characteristics, methods, and correlation with rectal temperature. Vet. J..

[44] Willits S. (2005). Infrared Thermography for Screening and Early Detection of Mastitis Infections in Working Dairy Herds. InfraMation Proc. ITC.

